# Developing R&D Portfolio Business Validity Simulation Model and System

**DOI:** 10.1155/2015/348369

**Published:** 2015-03-29

**Authors:** Hyun Jin Yeo, Kwang Hyuk Im

**Affiliations:** ^1^Division of Digital Contents, Dongseo University, 47 Jurye-ro, Sasang-Gu, Busan 617-716, Republic of Korea; ^2^Department of Electronic Commerce, Paichai University, 155-40 Baejae-ro, Seo-Gu, Daejeon 302-735, Republic of Korea

## Abstract

The R&D has been recognized as critical method to take competitiveness by not only companies but also nations with its value creation such as patent value and new product. Therefore, R&D has been a decision maker's burden in that it is hard to decide how much money to invest, how long time one should spend, and what technology to develop which means it accompanies resources such as budget, time, and manpower. Although there are diverse researches about R&D evaluation, business factors are not concerned enough because almost all previous studies are technology oriented evaluation with one R&D technology based. In that, we early proposed R&D business aspect evaluation model which consists of nine business model components. In this research, we develop a simulation model and system evaluating a company or industry's R&D portfolio with business model point of view and clarify default and control parameters to facilitate evaluator's business validity work in each evaluation module by integrate to one screen.

## 1. Introduction

Investing in the R&D (Research and Development) has been recognized not only as a key activity of companies and industries for one's sustainability, but also as a tool for expending market share and other purposes [1, 2]. Today, the R&D activity leads to registering domestic and international patent to protect one's knowledge property because knowledge has been recognized as a core asset especially in technology oriented company or industry. Therefore, patent has long been considered for representing a trade-off between incentives for innovation on one hand and competition in the market and diffusion of technology on the other [3].

Through the recognition to patent property as intangible asset, R&D evaluation has been widely studied in various industrial areas. However, almost all researches and studies are technology based evaluation and restricted to single R&D technology while APO noted that innovation shall be viewed from a broad perspective, not merely as technological improvement [4]. With respect to OECD and APO note, our former research utilized business model aspect approach to evaluate R&D validity by four modules with Osterwalder's nine business model components [2, 5].

In this research, we propose simulation model which is designed for systemize to facilitate a company or industry R&D competitiveness level evaluation in business model aspect with three control parameters and five default parameters.

## 2. Literature Review

### 2.1. Business Model

In business model research criteria, standard agreement on definition of business model is not settled while various previous researches have been conducted. Hedman and Kalling offer an outline for conceptual business models and propose it should include “customers and competitors, the offering, activities and organization, resources and factor market interactions.” Osterwalder et al.   defined business model as a “conceptual too containing a set of factors and their relationships and allows expressing the business logic of a specific company” [5, 6]. With review of the above two studies, proposed diversity of Morris et al. in the available definition of business model poses substantive challenges for delimiting the nature and components of a model and determining what constitutes a good model [7].

In this research, we suggest that business model could be comprehended as demonstrating how an organization purchases and sells goods and services as well as obtaining profits in the sense of the above literature reviews and via recent research trend to study the components of business model rather than definition [5, 8–10].

### 2.2. Business Model Component

In this research, with respect to recent research trend that concentrates not on definition but on components of business model, we adopted Osterwalder's nine business model components: value proposition, customer segments, channels, customer relationships, key activities, key resources, key partnerships, cost structure, and revenue stream, with four pillars: product, customer interface, infrastructure management, and financial aspects [5].

Table 1 shows text mined keywords for each BMC extracted from patents data in Korean automobile industry companies those are listed at Korean Automobile Industry Association. As one can see, text mined Korean automobile industry keywords which correspond to definition and general keywords which noted previous research [5] could be classified, and are industry oriented meaning keywords should be text mined for each industry because of industrial characteristics. Those keywords also support recent trend that concentrates on common components of business model, not on definition.

## 3. Methodology

### 3.1. Original Model

**Figure 1:**
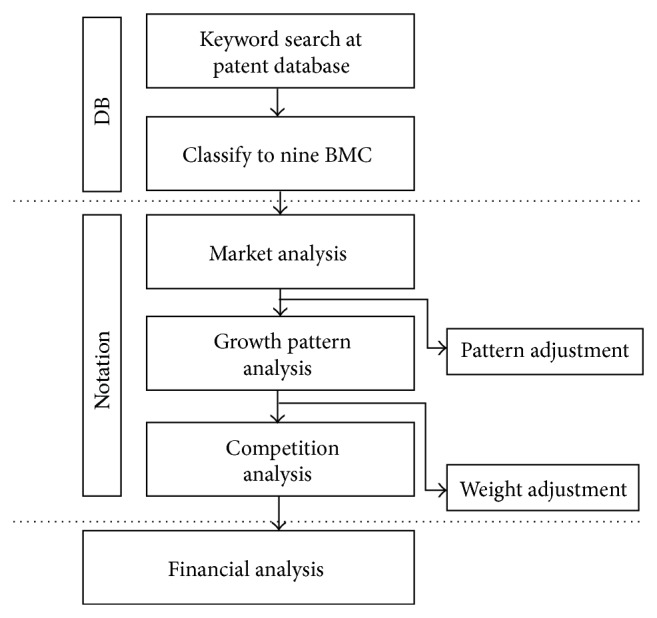
R&D business aspect evaluation model.

The simulation model in this research is established based on our former R&D business model evaluation model with four analysis steps: market, growth pattern, competition, and financial. Those have their own factors affecting company's or industry's R&D portfolio value which one can see in Figure 1 [2].

As one can see in Figure 1, original model already has three controllable parameters: pattern, current BMC value, and weight. In this research, the system adopted original model controllable parameters as system recommendation values because even the simulation could be independently operated; it has to set up scope of simulation control parameters to prevent extreme output by user faults.

The growth pattern analysis comes from the idea that market analysis simulates future market pie of analysis target by only linear growth pattern which is not suitable for real growth pattern. The diffusion of innovation theory and other growth pattern studies shows that market or customer growth follows similar technology market pattern [10].

Equation 1 shows original model growth pattern notation while 2 shows system recommendation log value of log function in growth pattern analysis. Equation 1 shows *t* year BMC_(*c*)*t*_ which means adjusted business value via log function pattern with analyzer input constant as a growth pattern adjustment. This step does not change *t* year simulated future value but adjusts process years for pattern that analyzer could adjust based on target's past pattern or future plan. In this research simulation model, the BMC_(*L*)_ in 2 which means log value which decides growth pattern curve shape, log or exponential, is recommended by system with comparing the first half period growth rate and last half one to reflect recent trend if it is growing up fast recently:

(1) BMCL=BMCM10−BMCM0BMCM010,BMCct=BMCM0+BMCL×BMCMtP,

(2) if  BMCM10BMCM6>BMCM5BMCM1 then  BMCL=BMCM10BMCM6else  BMCL=−BMCM5BMCM1.

### 3.2. Growth Rate (Recommendation)

Equation 3 notation illustrates the growth rate module of the original model using keyword CAGR (compounded annual growth rate) for each BMC. The notation shows growth rate for future business value which uses CAGR to each keyword and average of keywords CAGR in each BMC. In the model, we use past ten years patent data for BMP_(*M*)_ (business model pie) and calculate ten years future with that:

(3) Keyword  CAGR=×Beginning  Year  Patent  Quantity−1End  Year  Patent  Quantity  ×Beginning  Year  Patent  Quantity−11/(End  Year−BeginningYear+1)hhh−1×Beginning  Year  Patent  Quantity−1End  Year  Patent  Quantity,BMC  CAGR=AVERAGEKeyword  CAGR,BMCMt=BMCMt−1+BMCMt−1×BMC  CAGR.

In this research, we utilized industrial CAGR as a recommendation value of analysis of target's CAGR assuming that competitive environment leads benchmarking in one industry to competitors. In that, we assumed one company's R&D components growth would be mirrored by industry's one.

## 4. Development

Table 2 shows control and default parameters of the original and simulation model which are set up to systemize the model.

### 4.1. Control Parameters

To systemize the model, we set up three control parameters, BMC CAGR, log value, and BMC current value, which system user could control manually, and also made recommendation system for two control parameters, BMC CAGR and log value, with the methodologies above.

Control parameters are set up by diverse use case diagram of the system. BMC CAGR which controls target's BMC score weight assumes analysis target company has a plan to invest R&Ds which have some specific BMCs, and log value assumes target company expecting own R&D growth trend or long term plan of target while BMC current value considers missing patent data or analyzer's own supervision to BMC weight.

Different to two recommendation module adopted parameters, BMC current value is manual input interface control parameter. Equation 4 shows how the model decides target's R&D market scale with BMC and the portion of each BMC in market scale is core value of competition analysis because competition analysis compares analysis target's BMC portfolio to competitor's. In that, the current BMC value parameter should be handled carefully by analyzer and also hard to be automatically recommended

(4) Market  Pie≅∑Npatent,BMCM=∑BMCMNpatent,BMPM =∑tBMC(M)Value  Positiont  hhhhhhhhhhhhhh+Customer  Segmentst+Channelsthhhhhhhhhhhhhh+Customer  Relationshipsthhhhhhhhhhhhhh+Key  Activitiesthhhhhhhhhhhhhh+Key  Resourcest+Key  Partnershipsthhhhhhhhhhhhhh+ Cost  Structuret+Revenue  Streamt.

### 4.2. Default Parameters

Default parameters are barrier for control parameters to control extreme inputs from them which could cause unintentional results such as one BMC biased evaluation. Parameters in the simulation model are basically inherited from original model because basic reference data of simulation model comes from patent data in the original model. In that sense, default parameters restrict control parameters range. Firstly, the log value is restricted from −1 to +10 that makes presently possible growth graph. Secondly, we set up BMC weight summation as always 100% by utilizing comparative potion of BMC for BMC weight to compare to another company or industry.

### 4.3. Parameter Value Effect Diagram

**Figure 2:**
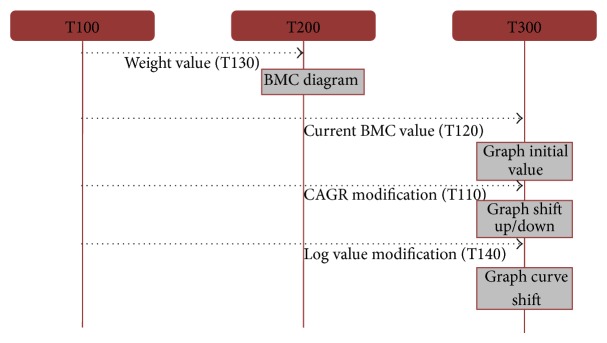
Parameter value effect diagram.

Since the simulation model is designed to be systemized, we also developed value effect diagram to analyze control parameter's effect on another parameter, value, and even screen.  Figure 2 is the BMC part of diagrams which illustrates that four values are generated from T100 module and give direct effect to two modules T200 and T300. The request and response diagram is utilized not only to define effect of values but also to facilitate understanding system design by developers.

## 5. Implementation and Limitation

**Figure 3:**
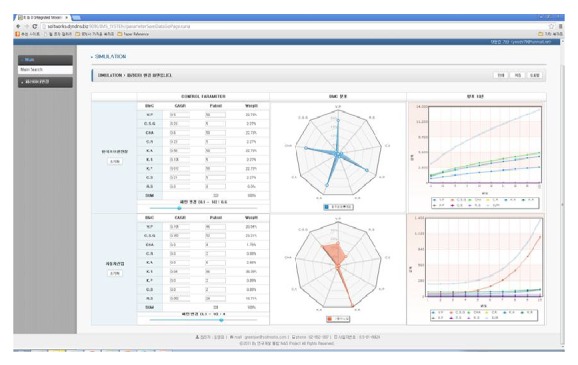
Simulation system.

The simulation model in this research is systemized with JAVA based platform as one can see in Figure 3. Figure 3 is prototype system screen of the system with past ten years Korean automobile industry patent data having 121,512 BMC keywords matching. The user interface consisted of three sectors, control parameter, BMC distribution, and growth pattern, which means one controllable sector and real time result sectors. The system interface is designed to show result in real time because we want to consider as many user needs as possible about assessment of one's R&D portfolio.

Although the model in this research considers diverse aspect and effects, the model has some limitations. Firstly, it could not reflect technical advance of one patent such as 4G telecommunication patent which could make huge advantage to owner. Hence, conversing one patent level evaluation and business model level evaluation would be necessary. Secondly, keywords which we used for this model are text mined from Korea automobile industry patent data. In that, to adopt this model for other industries, executing re-text mining target industry or auto-text mining system would be acceptable. Lastly, it is not suitable to company or industry having not enough patent data such as new established company or not technology oriented companies or industries.

Despite the limitations of this research, the model in this research has various potential implementations not only for company but also for industry or country level. Firstly, it could offer comparable value of company R&D activities which support decision making to R&D investment such as when, what, and how much one invest to R&D. The comparable value of R&D also support government to decide what industry or company she invest to maximize future knowledge property value. Furthermore, it could supplement previous technology based one patent level analysis with business model aspect even if one R&D has innovative factors but has no business value.

Although this study suggests not only notations for initial assessment model specialized in business model aspect but also simulation model for it, further studies in these criteria such as R&D process assessment and convergence system to technology based evaluation system are necessary because we think it is start-up trial adapting business model to technology based point of view.

## Figures and Tables

**Table 1:** BMC definition and Korean automobile industry patents data text mined keywords.

Pillar	BMC	Definition	Text mined keywords
Product	Value propositions	A value proposition is an overall view of a company's bundle of products and services that are of value to the customer.	Product improvement, convenient, safety drive, product satisfaction

Customer interface	Customer segments	The target customer is a segment of customers a company wants to offer value.	Passenger, driver, worker, user, customer
	Channels	A distribution channel is a means of getting in touch with the customer.	Circulation, logistics, transaction, network,
	Customer	The relationship describes the kind of link a company establishes between itself and the customer.	Community, service, customer management, interface, trust improvement

Infrastructure management	Key activities	The value configuration describes the arrangement of activities and resources that are necessary to create value for the customer.	Process, activity, productivity, development, problem solving
	Key resources	A capability is the ability to execute a repeatable pattern of actions that is necessary in order to create value for the customer.	Environment, support, resource, system, utility
	Key partnerships	A partnership is a voluntarily initiated cooperative agreement between two or more companies in order to create value for the customer.	Competitor, cooperation, partner, automobile industry, coalition

Financial aspects	Cost structure	The cost structure is the representation in money of all the means employed in the business model.	Cost decrease, cost reduction, fixed cost, variable cost, technology development cost
	Revenue stream	The revenue model describes the way a company makes money through a variety of revenue flows.	Benefit, profit, price, payment, rent, licens (“e” is omitted for licensing), fee

**Table 2:** Original model and simulation control and default parameters.

Segment	Analysis	Control parameters	Default parameters
Model	Market		Industrial BMC keywords
	Growth pattern		
	Competition	Current BMC value	Weight summation

Simulation	Market	BMC CAGR	
	Growth pattern	Log value (recommended)	Log range
	Competition	BMC current value (recommended)	Weight summation
